# Manuscript Architect: a Web application for scientific writing in virtual interdisciplinary groups

**DOI:** 10.1186/1472-6947-5-15

**Published:** 2005-06-16

**Authors:** Ricardo Pietrobon, Karen C Nielsen, Susan M Steele, Andreia P Menezes, Henrique Martins, Danny O Jacobs

**Affiliations:** 1 Division of Orthopaedic Surgery, Center for Excellence in Surgical Outcomes, Duke University Medical Center, Box 3094, Durham, NC 27710, USA; 2 Anesthesiology – Ambulatory Services, Duke University Medical Center, Box 3094 Med Ctr Durham, NC 27710, USA; 3 Anesthesiology – Ambulatory Services Box 3094 Med Ctr Durham, NC 27710, USA; 4 Hospital e Maternidade Angelina Caron, Rodovia do Caqui, 1150 – Km 1, Campina Grande do Sul, PR 83430, Brazil; 5 Iman Solutions, Center for Excellence in Surgical Outcomes, Duke University, USA; 6 Department of Surgery, Box 3704 Med Ctr Durham, NC 27710, USA

## Abstract

**Background:**

Although scientific writing plays a central role in the communication of clinical research findings and consumes a significant amount of time from clinical researchers, few Web applications have been designed to systematically improve the writing process.

This application had as its main objective the separation of the multiple tasks associated with scientific writing into smaller components. It was also aimed at providing a mechanism where sections of the manuscript (text blocks) could be assigned to different specialists. Manuscript Architect was built using Java language in conjunction with the classic lifecycle development method. The interface was designed for simplicity and economy of movements. Manuscripts are divided into multiple text blocks that can be assigned to different co-authors by the first author. Each text block contains notes to guide co-authors regarding the central focus of each text block, previous examples, and an additional field for translation when the initial text is written in a language different from the one used by the target journal. Usability was evaluated using formal usability tests and field observations.

**Results:**

The application presented excellent usability and integration with the regular writing habits of experienced researchers. Workshops were developed to train novice researchers, presenting an accelerated learning curve. The application has been used in over 20 different scientific articles and grant proposals.

**Conclusion:**

The current version of Manuscript Architect has proven to be very useful in the writing of multiple scientific texts, suggesting that virtual writing by interdisciplinary groups is an effective manner of scientific writing when interdisciplinary work is required.

## Background

Any reader of the scientific medical literature knows that research prose is a very specialized use of language, distant from the general intellectual prose. Unlike writing in the humanities, the single most important function of scientific writing is the transfer of exact information and explicitly stated ideas. The writer of scientific reports attempts to convey the most precise meaning, in a logically coherent order, and in as few words as possible. Despite the obvious importance and distinction of the scientific writing when compared to other types of writing, no previous articles have described software solutions specifically designed to facilitate the process of manuscript writing (scientific articles and grant proposals) in virtual interdisciplinary groups.

The fundamental purpose of scientific discourse is not only the presentation of information, but rather its communication. It does not matter how well authors might regard their own writing; what really matters is whether the large majority of the audience understands what the authors wanted to communicate. Achieving simplicity in research texts is a complex task, the failure to write the final manuscript being one of the most common reasons for completed research projects not being published in peer-review journals [1,2]. The reason for this complexity is usually misunderstood. Most people assume that the difficulties in scientific writing are inherent to the scientific concepts, data, and analysis. However, Gopen [3] has argued that complexity of thought does not necessarily lead to a difficult text. In our manuscript, we argue that a software solution may assist researchers in simplifying this task.

It has been shown that information is interpreted more easily and more uniformly if it is placed where most readers expect to find it [3]. These needs and expectations of readers affect the interpretation not only of tables and illustrations but also of the text itself. Scientific readers have relatively fixed expectations about where in the text structure of scientific manuscripts they will encounter particular concepts. If writers can become consciously aware of these locations, they can better control the degrees of recognition and emphasis a reader will give to the various pieces of information being presented.

Experienced researchers are aware of these expectations, but this skill is acquired usually after many failed attempts to learn the "unwritten rules" of scientific writing, which usually go far beyond the common IMRaD guidelines [4]. This underlying concept of reader expectation is perhaps most immediately evident at the level of the largest units of discourse, a unit of discourse being defined as anything with a beginning and an end (e.g., a clause, a sentence, a section, an article, etc.). A research article, for example, is generally divided into recognizable sections, sometimes labeled Introduction, Methods, Results and Discussion. When the sections are confused in situations where too much experimental detail is found in the Results section, or when discussion and results are mixed together, readers are often equally confused. If these structural expectations are continually violated, readers are forced to spend a considerable amount of effort to understand its structure, taking time way from simply understanding the underlying message. In our article, we argue that a software solution might assist researchers in meeting readers' expectations in terms of text structure and its order, therefore substantially improving the final transmission of information.

Existing approaches: To our knowledge, no previous applications have explored the ability to use a Web application to allow interdisciplinary virtual groups to work synchronously and asynchronously on the same manuscript. Currently, word processors are the most common tools used for scientific writing. Also more formally known as document preparation systems, word processors are computer applications used for the production (including composition, editing, formatting, and possibly printing) of any sort of viewable or printed material. Word processing was one of the earliest applications for the personal computer in office productivity, prior to the widespread use of the World Wide Web. Therefore, the text content is most often stored in local computers and then exchanged through e-mails. Although electronic files are efficient for texts produced by single writers, interdisciplinary collaborations require more sophisticated exchange systems.

The objective of this article is to describe the Web application Manuscript Architect, designed to assist virtual interdisciplinary groups in the writing of scientific manuscripts (research papers or grant proposals).

## Implementation

### Goals

The primary goal of the Manuscript Architect application is to simplify the process of scientific writing. Manuscript Architect divides the writing process into the following steps: (1) list main concepts (text blocks), (2) establish hierarchical order of text blocks, (3) connect text blocks, (4) ensure consistency across text blocks, (5) use previous examples of text blocks with a similar focus, (6) facilitate the translation when manuscripts are not written in the language used by the target audience (readers and journals).

### Design objectives

The overall objective of the Manuscript Architect project was to build a Web-based tool that would allow virtual groups to work on scientific manuscripts using synchronous and asynchronous methods. Before designing the application, we analyzed similar target applications in a diverse set of domains, including project management tools, text editors, tools for voice communication, and software tools for sharing of computer screens. Our search can be summarized into the following list of technical requirements that would be highly desirable for the application:

• Hierarchical navigation of text blocks, allowing authors to easily locate their current block within the entire manuscript. The concept of text blocks has been previously described by linguists such as Hoey [5]. A text block is a unit of text with a single focused content. In the Manuscript Architect application, we use the term text block to refer to a large unit of text, not necessarily related to the linguistic concept of text block. Similar approaches have been used by other applications such TuxCards [6].

• Fast screen upload for easy transition between text blocks

• Block text saving diversified among multiple application activities to avoid loss of text

• Primary author should be able to assign text blocks to co-authors and track their progress

• A full view of the manuscript should be available, displaying subsections as required

• Print to PDF or easy transference to commonly used electronic formats

• Word count by block texts and groups of block texts (e.g., abstract)

• Automated e-mails when a text block is assigned

• Color coding to identify the status of text blocks. For example, it should be clear for co-authors which text sections have been assigned to them and which sections have been completed

• Support for insertion of hyperlinks, figures, and other electronic files

• Robust security

• Scalability in the associated management system, including full compatibility with our existing project management application Research Manager

The Web application we have implemented to date meets all of these requirements. Other requirements will be met in our planned enhancements, as described in the Conclusion section below.

Software architecture for Manuscript Architect: The language of choice for the application development was JAVA, since it facilitates the integration of the Manuscript Architect application with the other existing applications in our suite [7]. We chose the classic life cycle development method [8], which includes the following steps: (1) the analysis and specification of pre-requisites using prototyping methods, (2) project, (3) implementation and unit testing, (4) system integration and testing, and (5) operation and maintenance.

**Figure 3:**
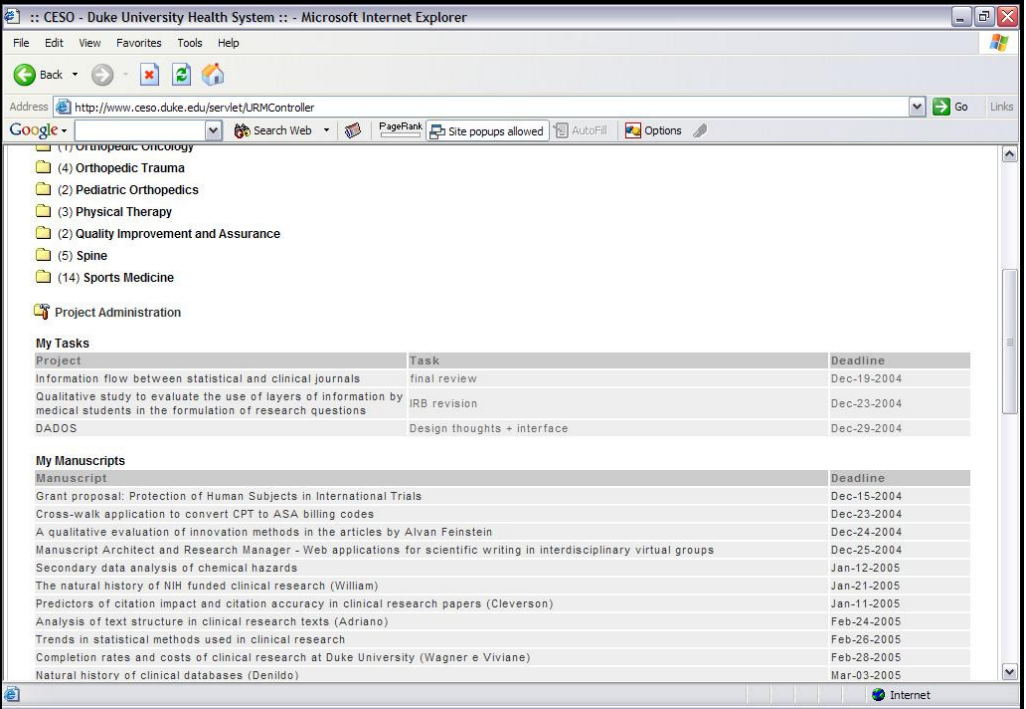
Manuscript Architect interface.

**Figure 4:**
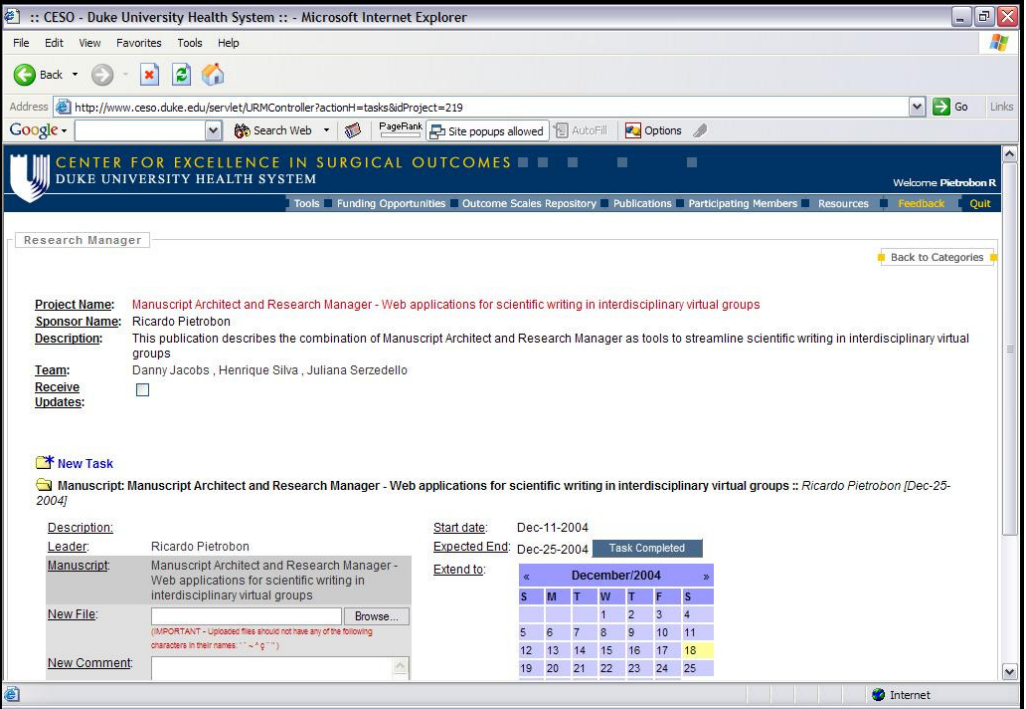
Manuscript Architect interface.

**Figure 5:**
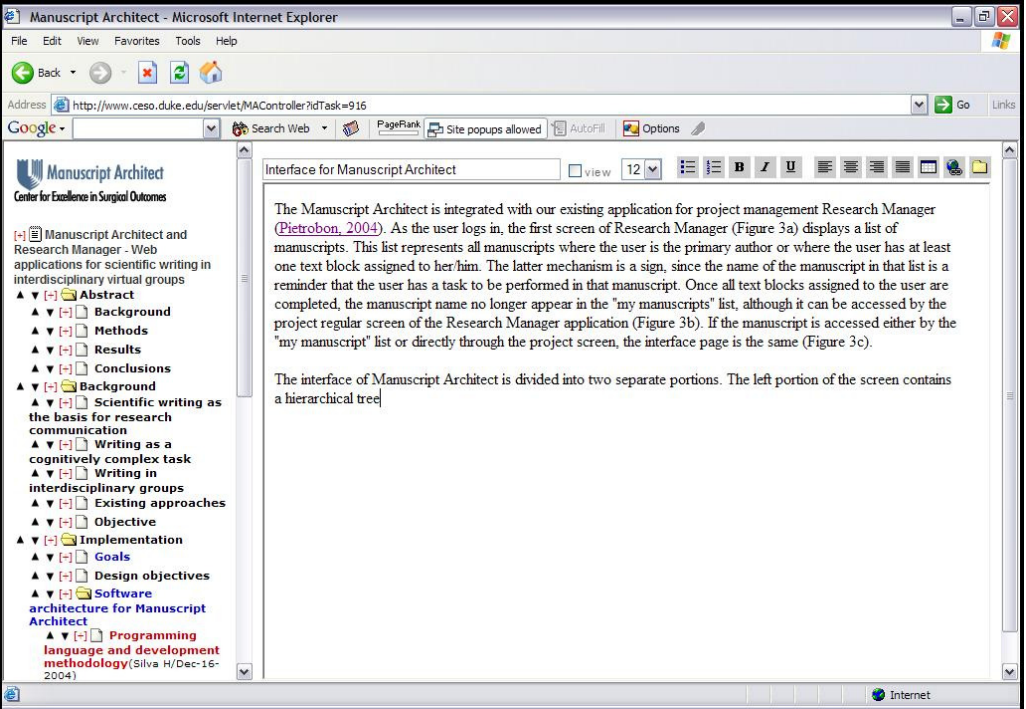
Manuscript Architect interface.

The Manuscript Architect application works integrated with Research Manager, an application developed by our group for project management [7]. As the user logs into Research Manager, the first screen of Research Manager (Figure 3) displays a list of manuscripts named "My Manuscripts". This list represents all manuscripts where the user is either the primary author or has at least one text block assigned to her/him. Having the name of the manuscript listed under "My Manuscripts" serves as a reminder that a writing task is to be performed in that manuscript. Once all text blocks assigned to the co-author are completed, the manuscript name no longer appears in the "My Manuscripts" list. It can, however, be accessed by co-authors through the Research Manager screen available for individual projects (Figure 4). Only project participants have password-protected access to the manuscript. If the manuscript is accessed either by the "My Manuscripts" list or directly through the project itself, the interface page for Manuscript Architect is the same (Figure 5).

### Interface

The interface of Manuscript Architect is divided into two separate portions. The left portion of the screen contains a hierarchical tree with all text blocks displayed in order of appearance. The hierarchical level is represented by the left indentation. In other words, a text block of level two is contained within the text block of level one right above it. The entire manuscript is contained under the root level. Text blocks where the user is currently working are highlighted in orange to facilitate identification. The title of each text block is identified by colors: red is a text block that has been assigned (the name of the assigned co-author and date of assignment appearing on the right side of the text block title; green is a text block that has been returned from a co-author to the primary author, and blue is a text block that has been marked as completed by the primary author. A button below the hierarchical tree assembles all text blocks into a single document, displayed on the right side of the screen.

The right portion of the Manuscript Architect application can display two types of screens. First, when the user has ownership over the text block, the block is editable and the window can be used as a regular word processor. A list of available editing tools include the ability to turn the title of the text block into a heading in the final manuscript, font size, bullets, numbered items, bold/italics/underlined, text alignment, table insertion, link insertion, and file insertion. Inserted pictures are displayed directly on the screen. Links to files, such as full-text documents, are displayed as hyperlinks. At the bottom of the screen a drop box allows the primary author to assign the text block to individual co-authors, save or remove the text block, and mark the text block as completed. Co-authors only have the save and completed options. Of importance, the text block is saved every time the user moves from one block to another. When the user does not have ownership of the text block, the right portion of the screen will be visible with read-only privileges. Primary authors have the ability to revoke the text block if necessary.

### Usability

Usability was evaluated at two distinct levels: (1) Formal usability analysis and (2) field observations. Formal usability analysis was conducted with ten different users with no previous experience with the Manuscript Architect. Five users had previous experience with scientific writing, while five were novice researchers.

Formal usability tests [9] followed a protocol where users were observed by one evaluator (RP) and had to complete scientific writing using the notes and previous examples. Users were free to ask questions at any point in time. Tasks included the writing of different portions of scientific articles, assigning different text blocks to different users, count words, observe the document in full-view, recover text blocks that had been initially assigned, insert links, insert documents (pdf files), and change font and alignment characteristics. Each participant answered a questionnaire at the end of the formal usability analysis with items about interface problems, missing features, and suggestions for overall improvement.

Field observations were comprised by observation of researchers using Manuscript Architect for the writing of scientific articles during an online writing workshop where over twenty different manuscripts were being written at the time by 14 different researchers (for a current list, please refer to ). Junior researchers include medical students, graduate students, residents, fellows, and junior faculty. During these workshops, Manuscript Architect is coupled with Voice over the Internet Protocol (VoIP) applications. Most of the manuscripts include co-authors from different disciplines located in at Duke University as well as other academic institutions in the U.S. and abroad. Similar to the formal usability tests, the evaluator (RP) took notes of problems encountered during the writing session regarding interface, missing features, and overall suggestions.

## Results

Manuscript Architect was designed to separate all activities involved in the production of a scientific text into a number of sequential steps. Following a sequence of steps facilitates the writing process since it eases the number of constraints that must be satisfied at one time and also increases the likelihood of satisfying any particular constraint. This sequence can be described as follows:

1. The first author designs the overall structure of the manuscript. This structure includes the inclusion of specific text blocks in a hierarchical order. For example, the first author may decide that the Introduction (level 1) should have the following text blocks under it (level 2): significance, knowledge lag, brief review of the status of knowledge, and objective. The words describing the central objective of each text block becomes its title.

2. Each text block is supplemented by a note explaining in detail the focus of the block. Notes are marked as meta-data, not being part of the main text of the manuscript. This explanation complements and extends the description already given by the title of the text block.

3. Each text block receives a previous example of a block with a similar focus. Similar to notes, previous examples are also marked as meta-data. Examples are chosen based on their structure rather than their topic.

4. Administrator assigns text blocks to users. Users are all co-authors of the manuscript, which has already been defined in the Research Manager application. Co-authors are chosen based on their expertise for the text block at hand. Therefore, a statistician might be assigned to the text block associated with statistical methods, while a medical student might be assigned a block where a literature review is required. When a text block is assigned, co-authors receive an automated e-mail. They are also notified of their text block assignment by a line under the "My Manuscripts" heading on the first page of the Research Manager application.

5. Co-authors write their text blocks in accordance with the note guidelines and the previous example. Once completed, co-authors mark the text block as "completed", which automatically returns the text block to the administrator.

6. Several iterations are made to ensure that the text block is adequately written. These iterations may be accomplished asynchronously or synchronously during writing workshops performed using VoIP (Voice of the Internet Protocol) tools.

7. When the text block is written in a language other than the language of the target journal, the first author might assign the block to the translator.

8. Once the block is translated, the text block is returned to the first author for revision. The version in the original language is kept in a separate meta-data field.

9. Once all text blocks are completed, the manuscript is formatted according to the guidelines of the target journal and submitted.

### Usability

The results for the formal usability analysis revealed that users were satisfied with the speed of the application, which they considered as an important factor in the transition from a word processor writing environment to a Web writing environment. Other characteristics were positively ranked by users in the formal usability analysis (Table 1). Two users requested an additional feature to highlight the title of the text block (Table 2). Results from the field analysis demonstrated that first authors were satisfied with the ability to assign text blocks to other investigators with greater expertise in certain areas. Many suggestions were made regarding the ability of track co-author's writing performance. This additional feature was judged particularly helpful in the tracking of graduate students. In response to their concern, we have added the following measures of performance: Total number of words written on the current day, average number of words written over the last seven days, and total number of words ever written by the researcher while using Manuscript Architect. A ranking of the maximum average number of words among all users was also added to the first page of the Research Manager application, from which all manuscripts are accessed.

**Table 1:** Satisfaction rating in formal usability analysis

Item construct*		**Evaluation rating**
MA speed is excellent	Strongly disagree	0/8
	Disagree	0/8
	Neutral	0/8
	Agree	1/8
	Strongly agree	7/8

MA is extremely easy to learn	Strongly disagree	0/8
	Disagree	0/8
	Neutral	2/8
	Agree	4/8
	Strongly agree	2/8

MA is extremely easy to use	Strongly disagree	0/8
	Disagree	0/8
	Neutral	1/8
	Agree	5/8
	Strongly agree	2/8

It is very easy to find the writing functions (e.g., assigning text blocks, viewing previous examples) associated with MA	Strongly disagree	0/8
	Disagree	0/8
	Neutral	1/8
	Agree	5/8
	Strongly agree	2/8

The navigation in MA is highly intuitive	Strongly disagree	0/8
	Disagree	0/8
	Neutral	0/8
	Agree	5/8
	Strongly agree	3/8

* MA = Manuscript Architect

**Table 2:** Free text recommendations and observations after formal and field usability analyses

**Suggestions for improvement of interface**
Highlight the title of the text block
**Missing features that should be added to MA**
Ability of track co-author's writing performance

**Suggestions for overall improvement of the MA application**
Translator features were not considered to provide adequate assistance in the process of converting texts to English
Use of non-scientific translators were deemed to add information noise to the scientific communication

**Features that you consider as an important additional value offered by MA in contrast with regular word processors**
Ability to assign text blocks to co-authors

Although we had high expectations regarding the possibility having a non-scientist translator converting text written by researchers whose first language was not the same as the target journal – most commonly English – the system was not efficient. The lack of efficiency resulted from (1) the difficulty in finding translators that were proficient in both the primary and target language and (2) the "communication noise" resulting from having a non-researcher translating research concepts. The translating tools were therefore only used when the first author was bilingual. In this situation, the system was effective since it allowed the first author and co-authors performing parallel tasks in the same manuscript. Although shared screen applications were also available during workshops, we have found that they are unnecessary.

## Discussion

To our knowledge, this is the first description of a Web application to facilitate the writing of scientific manuscripts by virtual interdisciplinary groups. Manuscript Architect simplifies the writing of scientific manuscripts by decomposing them into smaller units, thus making the writing task cognitively simpler. Although at this point Manuscript Architect has only been used within the Center for Excellence in Surgical Outcomes, the free distribution of its source code under the GNU Public License is expected to spread its use among other institutions.

Much of the difficulty of writing stems from the large number of constraints that must be concomitantly satisfied. In expressing a research idea the writer must consider at least four structural levels at once: (1) overall text structure, (2) paragraph structure, (3) sentence structure (syntax), and (4) word structure (spelling). Clearly, the attempt to coordinate all these requirements is a very difficult task which, over time, leads researchers to discouragement.

Another great difficulty for writers is maintaining connective flow. In other words, the relationship between ideas must be made clear while the idea must be expanded downward into paragraphs, sentences, words, and letters. Sometimes writers, and in particular the novice ones, become lost is the process of downward expansion and lose sight of the high-level relationships they originally wanted to express. Down sliding, the phenomenon of getting pulled into lower and more local levels of task processing is a very common problem in scientific writing. If a researcher decides to focus on accuracy in spelling and grammar, it will reinforce the natural tendency toward down sliding [10-18]. The enrichment provided by virtual peer-review is perhaps one of the most appealing areas in the field of virtual group writing. With the advent of new software tools, many of the tasks associated with scientific writing may be better distributed, in contrast with the now prevalent model of one researcher in charge of the manuscript while others simply review it for intellectual content. The socialization of this process has many consequences in terms of productivity and quality. From the perspective of peer-review, virtual group writing emphasizes the social construction of knowledge, a theoretical perspective characterized by the assertion that knowledge is created through social interaction [19]. Facilitating the exchange of information with other researchers will most likely improve quality, since it moves to an earlier stage a peer-review process that in the classic writing models is characteristically intensified only after the manuscript submission.

## Conclusion

Although Manuscript Architect was primarily designed for writing within interdisciplinary virtual teams, it can also be used in a variety of other circumstances such as: (1) use by single writers who would like to maintain their manuscript in an on-line environment while taking advantage of the stepwise approach of building a manuscript, and (2) educational writing workshops [20,21].

The main features planned for future versions of Manuscript Architect include integration with other tools previously developed by our group [7]. First, Manuscript Architect will be linked to literature maps, a method developed for structured literature reviews. This method will enhance the accuracy of literature citations as well as allow the literature search to run in parallel with other project activities. For example, a senior researcher can work on a text block of the discussion, while a junior researcher conducts an in-depth review of references to be later used in another text block of the same discussion. Second, links will be created between Manuscript Architect and the method of layers of information. The method of layers of information allows researchers without a formal graduate degree in statistics to understand complex statistical techniques by explaining them in a hierarchical fashion. Third, Manuscript Architect will be integrated with QuestForm, a Web application designed to formulate research questions based on existing data sets. The integration with QuestForm will allow for manuscripts to receive the description of databases, the variables used to answer the research question, ICD9 and CPT codes (when applicable), and tables and graphics resulting from the statistical analysis. Finally, text maps will be included to orient writers in the co-dependency across different text blocks. For example, if the Results section of a manuscript describes a certain patient outcome, a corresponding block should exist in the Methods section describing how the data describing that outcome was collected, validated, and analyzed. Text maps will enforce the checking of accurate links among different text blocks.

In conclusion, Manuscript Architect has proven to be a useful tool for the interdisciplinary collaboration among clinical researchers in academic centers. Future investigations should evaluate its role in undergraduate education as well as translational research.

## Availability and requirements

The Manuscript Architect application is available at . The Manuscript Architect application is distributed under the GNU General Public License. This license ensures that the source code can be freely distributed, modified, or even sold, as long as the source code is provided with every copy of the application. The source code for the application is available at no charge for download at 

## Abbreviations

VoIP: Voice of the Internet Protocol

GNU: GNU's Not Unix

ICD9: International Classification of Diseases, 9th Revision

CPT: Current Procedural Terminology

GPL: General Public License

## Competing interests

The author(s) declare that they have no competing interests.

## Authors' contributions

RP was primarily responsible for the application design, performed the usability testing, and drafted the manuscript

HM assisted in the application design, wrote the source code for the application, wrote the sections on software architecture, and reviewed the manuscript for intellectual content

AM assisted in the application design, assisted in the usability testing, wrote portions of the usability assessment, and reviewed the manuscript for intellectual content

KN, SS, and DJ assisted in the application design, assisted in the usability testing, and reviewed the manuscript for intellectual content

**Figure 1:**
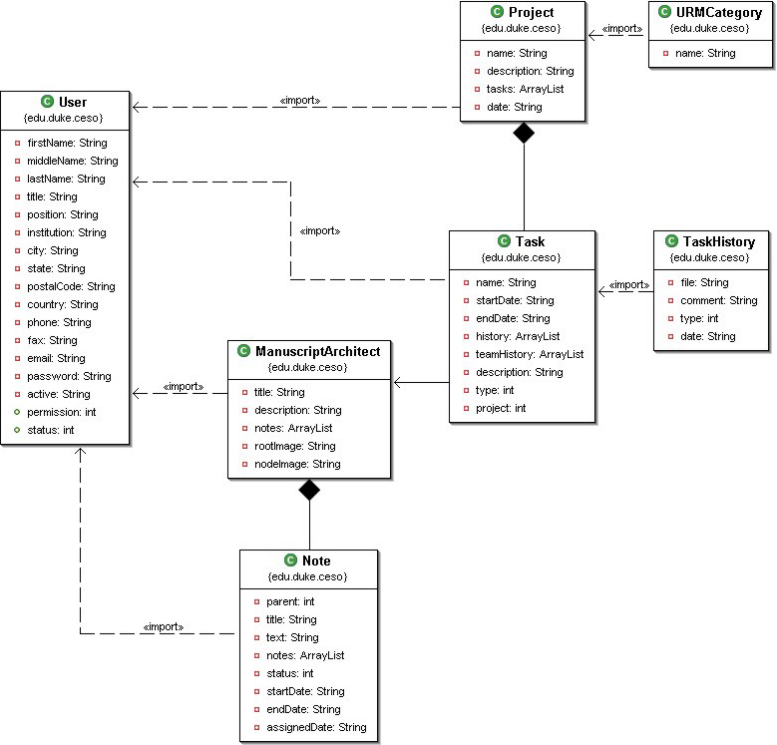
Class Components.

**Figure 2:**
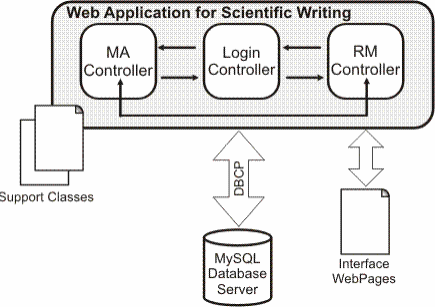
Manuscript Architect architecture.

## Pre-publication history

The pre-publication history for this paper can be accessed here:


