# Manuscript Architect: a web application for scientific writing in virtual interdisciplinary groups

**DOI:** 10.1186/1472-6947-7-21

**Published:** 2007-07-25

**Authors:** Ricardo Pietrobon, Karen C Nielsen, Susan M Steele, Andreia P Menezes, Henrique Martins, Danny O Jacobs

**Affiliations:** 1 Division of Orthopaedic Surgery, Center for Excellence in Surgical Outcomes, Duke University Medical Center, Box 3094, Durham, NC 27710, USA; 2 Anesthesiology – Ambulatory Services, Duke University Medical Center, Box 3094 Med Ctr Durham, NC 27710, USA; 3 Anesthesiology – Ambulatory Services Box 3094 Med Ctr Durham, NC 27710, USA; 4 Hospital e Maternidade Angelina Caron, Rodovia do Caqui, 1150 – Km 1, Campina Grande do Sul, PR 83430, Brazil; 5 Iman Solutions, Center for Excellence in Surgical Outcomes, Duke University, USA; 6 Department of Surgery, Box 3704 Med Ctr Durham, NC 27710, USA

After the publication of the above manuscript [1], the authors noticed that the 'Background' contained sentences from another publication [2]. This was an unintentional error. We did not intend to claim credit for work carried out by the author of the original publication. One of our co-authors was unaware that these sections could not be included verbatim without acknowledging their source. The authors would like to apologise for this oversight.

## Pre-publication history

The pre-publication history for this paper can be accessed here:


